# A Case of Severe Hepatitis in Infant Twins With COVID-19

**DOI:** 10.7759/cureus.41967

**Published:** 2023-07-16

**Authors:** Aaron L Heston, Varun Sharma, Taryn Johnson, Archana Anandakrishnan, Ashish Patel

**Affiliations:** 1 Pediatrics, Phoenix Children's Hospital, Phoenix, USA; 2 Medicine, University of Arizona College of Medicine, Phoenix, USA; 3 Pediatric Gastroenterology, Phoenix Children's Hospital, Phoenix, USA

**Keywords:** pediatric case with covid-19, liver fibrosis, pediatric gastroenterology, hepatology & gasrtroenterology, covid 19

## Abstract

We report a case of nine-month-old twins who presented with bright green diarrhea along with progressively worsening jaundice over one week. On initial evaluation, they were found to have significantly elevated liver enzymes, bilirubin, and alkaline phosphatase levels but without signs of liver failure. They were tested for multiple causes of liver injury including autoimmune and infectious etiologies, which were negative as well. Both twins were incidentally found to be positive for COVID-19 on testing per hospital protocol but did not have any respiratory symptoms. They were monitored closely during their hospital stay and showed clinical stability but with only slight improvement in abnormal lab levels. Ultimately, they were discharged with close outpatient follow-up. They demonstrated full resolution of all lab abnormalities and symptoms two months post discharge.

## Introduction

Severe acute respiratory syndrome coronavirus 2019 (SARS-CoV-2) causes COVID-19. This virus is most notable for its effects on the respiratory system, but it can present various disease phenotypes. A less common clinical finding is severe liver injury with substantially elevated liver enzymes, commonly associated with poorer clinical outcomes in adults [1-3]. This has been thought to be due to the localized inflammatory effects of the virus on the liver [3]. There has been extensive research on the effects of COVID-19 in the adult population, but research is more limited in the pediatric population. With the lack of research on the effects of SARS-CoV-2 on the pediatric population comes an incomplete understanding of the rarer effects the virus can cause in multiple organ systems. In this case, we present twin infants who initially presented with severe jaundice and elevated transaminases and were found to be positive for COVID-19.

## Case presentation

Nine-month-old dichorionic diamniotic male twins initially presented with a week of bright green diarrhea along with progressively worsening jaundice now encompassing the whole body. They were noted to be afebrile along with no other constitutional symptoms, and had no changes to their feeding. The parents described a mild diarrheal illness among others in the household that had spontaneously resolved in the week prior.

Their birth history was notable for delivery at 32 weeks due to maternal pre-eclampsia, which required monitoring in the NICU to work on oral feeding skills. While in the NICU, twin A developed mild necrotizing enterocolitis, which was managed conservatively with bowel rest and empiric antibiotics. Newborn screenings done on both twins revealed that Twin B was a carrier for cystic fibrosis, but a follow-up skin chloride test was negative. Neither twin required phototherapy at birth, and they had been meeting all developmental milestones and regularly attending primary care appointments.

They presented to the emergency department with extensive, whole-body jaundice and a pronounced scleral icterus. The rest of their physical examination and vital signs were normal for their age, and there were no neurological abnormalities. Their comprehensive metabolic panels (CMP) were both noted to have significantly elevated transaminases. Twin A had an aspartate transferase (AST) of 1134u/L and an alanine transferase (ALT) of 265u/L; twin B had an AST of 834u/L and an ALT of 263u/L. Twin A’s total bilirubin was 8.6mg/dL and direct was 6.0mg/dL, while twin B’s total bilirubin was 6.8mg/dL and direct was 4.7mg/dL. The complete blood count (CBC) was normal for their age and showed no leukocytosis, anemia, or other evidence of hemolysis. Electrolyte values and creatinine were normal. Though they exhibited no respiratory symptoms, a respiratory virus panel was obtained per hospital protocol and showed they were both positive for SARS-CoV-19 by polymerase chain reaction test. Other inflammatory markers, including C-reactive protein, erythrocyte sedimentation rate, and procalcitonin, were unremarkable. A right upper quadrant ultrasound showed no cholelithiasis or biliary obstruction. Coagulation labs, as a representation of hepatic function, were normal. With stable vital signs and no other remarkable exam findings, they were admitted to the hospital for further workup.

Hepatology was consulted and recommended labs to test for infectious and autoimmune liver pathology: anti-nuclear antibody, anti-liver kidney (LK)/F-actin antibody, urine organic acids, plasma amino acids, hepatitis panel, iron, ferritin, epstein-Barr virus, cytomegalovirus, herpes simplex virus, human herpes virus 6, and adenovirus labs were all normal. A blood smear obtained to assess hemolysis was found to be unremarkable.

On hospital day three, a liver biopsy was performed on both twins. Their results were similar and demonstrated stage two portal and peri-portal fibrosis with lobular cholestasis and hepatocellular necrosis. These findings were grossly consistent with cholestatic liver disease. There was no excessive iron or copper disposition and no pathologic findings of alpha-1 antitrypsin deficiency. Genetics was consulted, and the Cincinnati Liver Disease Panel was obtained, which was negative for the most common genetic causes of hereditary liver disease, including progressive familial intrahepatic cholestasis. Urine bile acids and carbohydrates were analyzed and showed negative results for deficiencies in carbohydrate metabolism.

Transaminase and bilirubin levels were serially monitored throughout the hospitalization (Table 1), which moderately improved after eight days. On the day of discharge, twin A had an AST of 941u/L, and twin B had an AST of 549u/L. The patients were discharged and scheduled for outpatient follow-up. After two months of close monitoring, the transaminases had normalized, and neither twin showed signs of jaundice.

**Table 1:** Selected markers of liver function and cholestasis at the time of admission, discharge, and follow-up AST: Aparte transferase, ALT: Alanine transferase

Markers	Twin A			Twin B		
Lab test (reference values)	Admission	Discharge (hospital day 8)	Follow-up appointment (60 days after discharge)	Admission	Discharge (hospital day 8)	Follow-up appointment (60 days after discharge)
							AST (15-37 u/L)	1134	941	45	834	549	40
ALT (30-65 u/L)	265	195	23	263	204	24
Total bilirubin (<0.8 mg/dL)	8.6	8.2	0.2	6.8	6.2	0.2
Direct bilirubin (0.0-0.4 mg/dL)	6	5.7	0.07	4.7	4.4	0.08
Alkaline phosphatase (146-477 u/L)	1323	880	0.2	963	483	270

## Discussion

While hepatic injury is a known side effect of COVID-19 infection, this case offers a novel presentation of COVID-19-associated liver disease in twin infants. Reports have shown that acute liver injury (ALI) occurs in 20% to 30% of adults hospitalized with COVID-19, though it is most commonly mild transaminitis that spontaneously resolves [4]. A recent case series demonstrated similar findings in the pediatric population, showing that COVID-19 infection combined with an ALT >40u/L was associated with more severe clinical outcomes, including ICU admission and kidney dysfunction. However, in this study, there were so few patients with ALT > 200u/L (5x the upper limit of normal) that their outcomes were not measured [5]. Both patients presented in this case had ALT levels > 200u/L but had a relatively benign clinical course without any evidence of kidney dysfunction.

A more recent pediatric case report showed a similar presentation in a 30-day-old female infant with a severe elevation of both AST and ALT without liver failure [6]. The patient had a course almost identical to our patients and recovered with conservative measures. While hepatic insult is also known to occur with multisystem inflammatory syndrome in children (MIS-C) [7], our patients did not meet any of the criteria for this diagnosis (fever for over 24 hours, laboratory evidence of inflammation, multisystem organ involvement, no alternative diagnoses, and recent SARS-CoV-2 infection, all of which must be met).

This case is also unique due to the relatively benign disease phenotype present at the time of admission. The COVID-19 infection typically presents with fevers, breathing difficulties, or cough [8], while hepatic dysfunction in infants often results in poor feeding and lethargy [9]. None of these symptoms were present in this case, and it was only their visually striking jaundice that prompted the parents to present for medical evaluation.

An aggressive genetic workup was undertaken because of their age and the fact that they are twins. Both twins had heterozygous (single) variants of uncertain significance in ABCC2 and EPHX1. The ABCC2 is associated with Dubin-Johnson syndrome [10] and codes for epoxide hydroxylase 1 (EPHX1), which is broadly expressed in the liver [11]. Both genes are associated with autosomal recessive conditions for which a second variant was not identified. Both twins had a heterozygous variant in UGT1A1, a risk allele for Gilbert only when it is homozygous [12]. Twin B was previously known from newborn screening as a carrier of the R117H gene, associated with cystic fibrosis [13] but he had a negative sweat chloride test. While these genetic results were all largely normal, this is a novel presentation of genetic investigation in a case of pediatric COVID-19-associated hepatitis. These clinically normal results suggest that the severity of the liver injury was solely due to COVID-19 infection as opposed to genetic predisposition.

## Conclusions

Despite the scale of the global pandemic, COVID-19 is still a relatively new and evolving pathogen with a wide variety of effects that are being uncovered as more research is done. The pediatric population has experienced varying effects from the virus that differ from those seen in the adult population. While there are few published reports of liver injury in the pediatric population secondary to COVID-19 infection, this case shows that liver pathology should be considered and evaluated in pediatric patients with COVID-19 infection.

## References

[1] Youssef M, H Hussein M, Attia AS (2020). COVID-19 and liver dysfunction: a systematic review and meta-analysis of retrospective studies. J Med Virol.

[2] Bloom PP, Meyerowitz EA, Reinus Z (2021). Liver biochemistries in hospitalized patients with COVID-19. Hepatology.

[3] Wu H, Liu S, Luo H, Chen M (2021). Progress in the clinical features and pathogenesis of abnormal liver enzymes in coronavirus disease 2019. J Clin Transl Hepatol.

[4] Phipps MM, Barraza LH, LaSota ED (2020). Acute liver injury in COVID-19: prevalence and association with clinical outcomes in a large U.S. cohort. Hepatology.

[5] Perez A, Cantor A, Rudolph B (2021). Liver involvement in children with SARS-COV-2 infection: two distinct clinical phenotypes caused by the same virus. Liver Int.

[6] Palpacelli A, Martelli G, Lattanzi B, Volpini A, Cazzato S (2021). Severe hypertransaminasemia during mild SARS-CoV-2 infection: a pediatric case report and literature review. Pediatr Investig.

[7] Cantor A, Miller J, Zachariah P, DaSilva B, Margolis K, Martinez M (2020). Acute hepatitis is a prominent presentation of the multisystem inflammatory syndrome in children: a single-center report. Hepatology.

[8] Liu X, Tang J, Xie R (2020). Clinical and epidemiological features of 46 children <1 year old with coronavirus disease 2019 in Wuhan, China: a descriptive study. J Infect Dis.

[9] Sgouropoulou V, Vargiami E, Kyriazi M, Papadimitriou E, Agakidis C, Zafeiriou D (2021). Transient severe liver injury: a unique presentation of COVID-19 disease in a pediatric patient. Pediatr Infect Dis J.

[10] Keitel V, Nies AT, Brom M, Hummel-Eisenbeiss J, Spring H, Keppler D (2003). A common Dubin-Johnson syndrome mutation impairs protein maturation and transport activity of MRP2 (ABCC2). Am J Physiol Gastrointest Liver Physiol.

[11] Liang SH, Hassett C, Omiecinski CJ (2005). Alternative promoters determine tissue-specific expression profiles of the human microsomal epoxide hydrolase gene (EPHX1). Mol Pharmacol.

[12] Kadakol A, Ghosh SS, Sappal BS, Sharma G, Chowdhury JR, Chowdhury NR (2000). Genetic lesions of bilirubin uridine-diphosphoglucuronate glucuronosyltransferase (UGT1A1) causing Crigler-Najjar and Gilbert syndromes: vorrelation of genotype to phenotype. Hum Mutat.

[13] Thauvin-Robinet C, Munck A, Huet F (2009). The very low penetrance of cystic fibrosis for the R117H mutation: a reappraisal for genetic counselling and newborn screening. J Med Genet.

